# Randomized clinical trial of ultrasonic scissors versus conventional haemostasis to compare complications and economics after total thyroidectomy (FOThyr)

**DOI:** 10.1002/bjs5.2

**Published:** 2017-05-09

**Authors:** C. Blanchard, F. Pattou, L. Brunaud, A. Hamy, M. Dahan, M. Mathonnet, C. Volteau, C. Caillard, I. Durand‐Zaleski, E. Mirallié, the FOThyr Group, V.‐P. Riche, S. Mucci, C. Nominé, R. Caiazzo, J. M. Prades, G. Landecy, H. P. Dernis, J. C. Lifante, F. Sebag, F. Jegoux, E. Babin, A. Bizon, F. Espitalier

**Affiliations:** ^1^ Clinique de Chirurgie Digestive et Endocrinienne Centre Hospitalier Universitaire (CHU) de Nantes Nantes France; ^2^ Département Promotion Délégation à la Recherche Clinique et à l'Innovation Nantes France; ^3^ Chirurgie Générale et Endocrinienne, CHU Lille Université de Lille Lille France; ^4^ Service de Chirurgie Digestive, Hépato‐Biliaire et Endocrinienne CHU Nancy – Hôpital de Brabois Nancy France; ^5^ Chirurgie Digestive et Endocrinienne CHU Angers Angers France; ^6^ Chirurgie Thoracique, Pôle Voies Respiratoires CHU de Toulouse – Hôpital Larrey Toulouse France; ^7^ Chirurgie Digestive, Générale et Endocrinienne CHU de Limoges – Hôpital Dupuytren Limoges France; ^8^ Assistance Publique – Hôpitaux de Paris Unité de Recherche Clinique en Économie de la Santé d'Île‐de‐France Hôpital de l'Hôtel‐Dieu Paris France; ^9^ Département Partenariats et Innovation Cellule Innovation, Délégation à la Recherche Clinique et à l'Innovation Nantes; ^10^ Chirurgie Digestive et Endocrinienne Centre Hospitalier Universitaire (CHU) Angers Angers; ^11^ Service de Chirurgie Digestive Hépato‐Biliaire et Endocrinienne, Nancy – Hôpital de Brabois Nancy; ^12^ Chirurgie Générale et Endocrinienne CHU Lille, Université de Lille Lille; ^13^ Oto‐Rhino‐Laryngologie (ORL) et Chirurgie Cervico‐Faciale et Plastique CHU Saint‐Etienne – Hôpital Nord Saint‐Etienne; ^14^ Chirurgie Digestive CHU de Besançon – Hôpital Jean Minjoz Besançon; ^15^ Service ORL et Chirurgie Cervico‐Faciale Centre Hospitalier du Mans Le Mans; ^16^ Chirurgie Générale, Endocrinienne, Digestive et Thoracique Centre Hospitalier Lyon‐Sud Pierre Bénite; ^17^ Chirurgie Générale Assistance Publique des Hôpitaux de Marseille – Hôpital de La Conception Marseille; ^18^ Service ORL et Chirurgie Maxillo‐Faciale CHU de Rennes – Hôpital Pontchaillou Rennes; ^19^ ORL et Chirurgie Cervico‐Faciale CHU de Caen Caen; ^20^ ORL et Chirurgie Cervico‐Faciale CHU d'Angers Angers; ^21^ Service ORL CHU de Nantes Nantes

## Abstract

**Background:**

The benefits of single‐use ultrasonic scissors in thyroid surgery are still debated. Although this device has been shown to reduce operating time compared with conventional haemostasis, its cost‐effectiveness has never been demonstrated. The aim of this study was to evaluate the efficacy, cost‐effectiveness and safety of ultrasonic scissors for total thyroidectomy.

**Methods:**

This was a prospective, randomized, multicentre trial conducted at 13 hospital sites. The primary endpoint was the percentage of patients with hypocalcaemia (serum calcium level below 2 mmol/l) on day 2. Secondary endpoints included postoperative complications and costs, with calculation of incremental cost differences and cost‐effectiveness ratios.

**Results:**

In total, 1329 patients who underwent total thyroidectomy were included in the analysis: 670 were randomized to treatment with ultrasonic scissors and 659 to conventional haemostasis. There was no difference between groups in the rate of complications, including hypocalcaemia on day 2 (19^.^7 per cent in ultrasonic scissors group versus 20^.^3 per cent in conventional haemostasis group; P = 0·743). Median operating times were significantly shorter with ultrasonic scissors (90 versus 100 min with conventional haemostasis; P < 0·001). Total mean(s.d.) direct costs at 6 months were €4311(1547) and €4011(1596) respectively (P < 0·001).

**Conclusion:**

Ultrasonic scissors were no more clinically effective than conventional haemostasis, but use of these devices was more costly. Registration number: NCT01551914 (http://www.clinicaltrials.gov).

## Introduction

Thyroidectomy is a common surgical procedure that is generally considered very safe, with small risks of hypoparathyroidism, recurrent laryngeal nerve (RLN) injury and compressive haematomas. Large case series suggest that rates of these complications are: haematoma, 1·2–2·1 per cent^1^, ^2^, ^3^, ^4^; transient hypoparathyroidism, 6·4–35·2 per cent^1^, ^2^, ^4^, ^5^, ^6^; permanent hypoparathyroidism, 0·9–6·3 per cent^1^, ^2^, ^3^, ^5^, ^6^; transient RLN injury, 0·36–3·9 per cent^1^, ^2^, ^3^, ^4^, ^5^; and permanent RLN injury, 0·7–1·4 per cent^1^, ^2^, ^3^, ^5^. Short‐term complications increase length of stay in hospital, the number of consultations, diagnostic tests and treatments needed, and overall cost. Permanent RLN palsy and hypoparathyroidism significantly impair patients' quality of life.

Ultrasonic scissors (US) use mechanical vibrations to cut and coagulate tissue simultaneously. As haemostasis is generated at lower temperatures, they are thought to cause less lateral thermal tissue injury than traditional electrocautery. The single‐use device, HARMONIC FOCUS^®^ (Ethicon Endo‐Surgery, Johnson & Johnson, Cincinnati, Ohio, USA), has been evaluated in a number of clinical studies with discordant results. Although it is generally acknowledged that use of US reduces operating time^7^, ^8^, ^9^, ^10^, the impact on postoperative complications is unclear. Some studies have reported lower rates of complications, whereas others failed to demonstrate benefits over conventional haemostasis (CH)^8^, ^9^, ^10^, ^11^, ^12^, ^13^, ^14^. It is also important to assess the cost‐effectiveness of such a device. The FOThyr RCT was designed to evaluate the 6‐month clinical efficacy and cost‐effectiveness of US (HARMONIC FOCUS^®^) compared with CH for thyroidectomy.

## Methods

The FOThyr study was a prospective, multicentre, single‐blind trial, with patients enrolled at 13 sites in France. All patients, aged 18–80 years, scheduled to undergo total thyroidectomy were eligible for enrolment in the trial if they had Graves' disease, toxic or non‐toxic thyroid goitre, or any thyroid nodule requiring total thyroidectomy via a cervical incision. Exclusion criteria were: thyroid cancer (known or suspected before operation based on ultrasound or cytological assessment, in order to avoid lymph node dissections (LNDs) associated with thyroidectomy); a calcitonin level exceeding 30 pg/ml; planned partial thyroidectomy; abnormal motility of vocal cords; substernal goitre (more than 3 cm below the sternal notch); surgery using videoscopy; and any history of cervical surgery. Preoperative serum levels of calcium, phosphorus, calcitonin, thyroid‐stimulating hormone and albumin were obtained for all patients. Preoperative vocal cord examination was undertaken only in patients who complained of a voice disturbance.

The protocol was reviewed and approved by a regional ethics committee (Comité de Protection des Personnes Ouest IV; 58/2012) and by the Commission Nationale de l'Informatique et des Libertés (1170319). The study was performed in accordance with the Good Clinical Practice Guidelines and the Declaration of Helsinki. All patients provided written informed consent before randomization, without any stipend. The study is registered at http://ClinicalTrials.gov (NCT01551914).

Patients were assigned randomly in a 1 : 1 ratio to the US or the CH arm of the trial, using block randomization, stratified by site. Allocation of treatment arm was done the day before surgery, according to a computer‐generated list. Only surgical staff members were aware of the allocated group.

### Procedures

In the experimental arm, haemostasis was performed using the US device. In the reference (CH) arm, the choice of devices was left to the discretion of investigators, depending on their usual practice involving clips, ligatures, monopolar and/or bipolar coagulation. All thyroidectomies were performed according to the same protocol. A transverse cervical incision was used, with a midline opening of the fascia and division of infrahyoid muscles as necessary. Vessels of the upper pole were divided, with preservation of the superior laryngeal nerve and all parathyroid glands whenever possible. RLNs were visualized at least for the last centimetre of their course. Postoperative drainage was left to the surgeon's discretion. Thyroid hormone replacement was introduced on postoperative day 1. All surgeons had experience in thyroid surgery and were familiar with both US and CH.

### Outcomes and evaluations

The primary endpoint was the percentage of patients with postoperative hypocalcaemia (serum calcium level below 2 mmol/l corrected for albumin level) on postoperative day 2. Calcium and albumin measurements were carried out in the laboratories of local hospitals. Secondary endpoints included postoperative morbidity (bleeding, RLN injury (immediate and permanent) and permanent hypocalcaemia) and postoperative pain. Bleeding was defined as the occurrence of a compressive haematoma requiring revisional surgery. RLN function was evaluated by vocal cord examination using transnasal endoscopy, carried out before hospital discharge and at 6 months after surgery, if abnormal postoperative motility had been identified. Permanent hypocalcaemia was defined by a serum calcium level below 2 mmol/l (corrected for albumin level) at 6 months. Pain was evaluated at 4 h, 18–24 h and 2 days after surgery, using a numeric pain rating scale and use of analgesic drugs; this information was collected by a ward nurse blinded to the group allocation. Clinical examination was performed by the surgeon during the hospital admission to detect haematomas, dysphonia and/or swallowing disorders. Adverse events were recorded in a prospectively developed database and graded using the Common Terminology Criteria for Adverse Events, version 3.0^15^.

The prospective economic evaluation was concurrent with the FOThyr randomized trial, and performed in accordance with the CHEERS statement^16^. The analysis was conducted from the healthcare perspective. Procedure costs were obtained using a bottom‐up microcosting approach that identified all relevant cost components of the surgical procedure and valued each component for all individual patients, using duration of surgery (time from skin incision to wound closure), staff, medical devices and type of operating room as variables. All consultations, tests, drugs and days of sick leave within 6 months after surgery were included. The duration of operating room occupancy was measured from the time the patient entered the room to the end of cleaning of the room.

Cost of the medical devices was the manufacturer's price. Staff costs were estimated from gross salaries and operating costs from the hospital accounting systems. Costs of hospital stays were estimated using the average national cost for each patient's diagnosis‐related group, adjusted for actual length of hospital stay and resources used during the hospital stay. Readmissions and non‐hospital costs were based on tariffs of the French healthcare system. Total costs for each group were calculated by summing costs for each patient. All costs are reported in euros (2014) and not discounted. Purchasing parity power (1€ = 1·28 US $) was based on the Organisation for Economic Co‐operation and Development 2015 guidelines (http://www.oecd-ilibrary.org/economics/data/agregats-des-comptes-nationaux/ppa-et-taux-de-change_data-00004-fr). Health outcomes were valued in terms of adverse events at 2 days and 6 months. A cost‐effectiveness analysis was conducted to estimate incremental costs per adverse event averted with US device compared with CH over a 6‐month period.

### Statistical analysis

Assuming a 25 per cent incidence of immediate hypocalcaemia and a significance level of 5 per cent, it was calculated that 648 patients per arm should be analysed to demonstrate a 30 per cent reduction of hypocalcaemia in the US group with a power of 80 per cent. As recommended in the International Council for Harmonization guidelines, two‐sided tests were used for all analyses (E9 Statistical Principles for Clinical Trials). To anticipate possible withdrawals of consent before surgery and lack of availability of the primary endpoint, it was planned to randomize 1350 patients.

Categorical variables are expressed as counts and percentages, and quantitative variables as mean(s.d.) and/or median (i.q.r.). Primary and secondary endpoints are described with their 95 per cent confidence intervals. Statistical significance was set at 0·050 (2‐sided).

All analyses were performed in the full analysis set (FAS) population, including all randomized patients who underwent surgery and who met the inclusion criteria. For primary endpoint and economic analyses, sensitivity analyses were done in the per‐protocol (PP) population. The PP population included all patients in the FAS without major protocol violation.

A generalized linear mixed model was used to compare the rates of postoperative complications and the effects of baseline pathology (euthyroidism or hyperthyroidism) and LND. Only permanent hypocalcaemia was analysed using a non‐inferiority approach. Non‐inferiority of US was demonstrated if the lower limit of the 95 per cent c.i. of the difference between CH and US was greater than −1 per cent.

For economic analysis, differences in costs and outcomes were tested by standard parametric or non‐parametric tests (Student's *t* or Mann–Whitney *U* test), as appropriate. Incremental cost difference and cost‐effectiveness ratio were calculated directly. Statistical significance for differences among *a priori* comparisons was set at *P* = 0·050 (2‐sided). The robustness of the cost‐effectiveness analysis results was explored by both deterministic and probabilistic sensitivity analyses, using non‐parametric bootstrap with 5000 replications.

Statistical analyses were performed with SAS^®^ software versions 9.2 and 9.3 (SAS Institute, Cary, North Carolina, USA).

## Results

**Figure 1:**
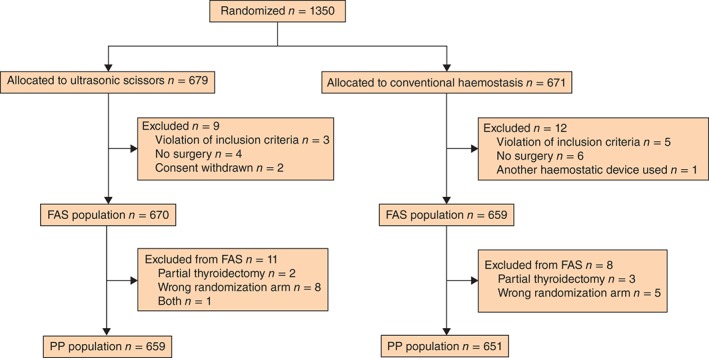
CONSORT flow chart for the trial. FAS, full analysis set; PP, per protocol

From March 2012 to June 2014, 1350 patients were enrolled in the study at 13 sites, with 679 randomized to the US arm and 671 to the CH arm (*Fig*.  1). Ultimately, the FAS comprised data for 1329 patients, 670 and 659 in the US and CH arm respectively. For sensitivity analyses, 19 patients were excluded from the PP population because of major deviations from the protocol: partial thyroidectomy (5), incorrect randomization arm (13) and both (1).

Baseline characteristics of the study group are summarized in Table 1. The two groups were well matched. Histopathological examination revealed that 21·9 per cent of patients had thyroid cancer, mostly of the papillary type. These patients were included in the analysis as thyroid cancer was not known or suspected before operation. LND (central in all patients) was performed 29 patients (2·2 per cent), with a median of 5 (i.q.r. 1–11) nodes harvested.

**Table 1:** Patient characteristics, procedures and histopathological data

	Ultrasonic scissors (n = 670)	Conventional haemostasis (n = 659)	Total (n =1329)
Baseline data			
Age (years)*	51·1 (40·9–61·9)	51·3 (40·0–61·2)	51·2 (40·9–61·4)
Sex ratio (F : M)	533 : 137	529:130	1062:267
BMI (kg/m^2^)*	25·5 (22·8–29·4)	25·9 (22·4–29·4)	25·6 (22·7–29·4)
TSH (µunits/ml)*	0·78 (0·25–1·44) (n = 635)	0·82 (0·31–1·53) (n = 615)	0·80 (0·27–1·48) (n = 1250)
Thyroid function			
Euthyroidism	502 (79·1)	493 (80·2)	995 (79·6)
Hyperthyroidism	133 (20·9)	122 (19·8)	255 (20·4)
Missing	35	44	79
Type of surgery and histopathological data			
Surgical procedure			
TT	658 (98·4)	639 (97·3)	1297 (97·8)
TT + LND	11 (1·6)	18 (2·7)	29 (2·2)
Missing	1	2	3
Thyroid cancer	150 (22·4)	141 (21·4)	291 (21·9)
Papillary	148	134	282
Other	2	7	9
Weight of thyroid gland (g)*	36 (22–54)	34 (21–58)	35 (22–57)

Values in parentheses are percentages unless indicated otherwise;

* values are median (i.q.r.). TSH, thyroid‐stimulating hormone; TT, total thyroidectomy; LND, lymph node dissection.

### Complications

The rate of hypocalcaemia on day 2 was 20·0 per cent (266 patients): 19·7 per cent (132) in the US arm and 20·3 per cent (134) in the CH arm. The between‐arm difference (mean 0·7 (95 per cent c.i. –3·5 to 5·0) per cent) was not statistically significant (P = 0·743). There was no significant difference in rates of other complications between the two arms (Table 2). With regard to the incidence of permanent hypocalcaemia, the difference between CH and US arms (mean −0·9 (−2·4 to 0·6) per cent) did not confirm superiority of the US technique.

**Table 2:** Early and late complications

	Ultrasonic scissors (n = 670)	Conventional haemostasis (n = 659)	Total (n =1329)	P §
Intraoperative complications				
Any complication	19 (2·8)	5 (0·8)	24 (1·8)	0·008
Device‐related complications†	2 (0·3)	0 (0)	2 (0·2)	
Dysfunction of device	14 (2·1)	0 (0)	14 (1·1)	
Thyroidectomy could not be performed	4 (0·6)	3 (0·5)	7 (0·5)	
Postoperative pain*				0·241
4 h	3 (1–4) (n = 618)	3 (1–4) (n = 614)	3 (1–4) (n = 1232)	
18–24 h	2 (0–3) (n = 624)	2 (0–3) (n = 621)	2 (0–3) (n = 1245)	
Day 2	1 (0–2) (n = 574)	1 (0–2) (n = 563)	(0–2) (n = 1137)	
Early postoperative complications				
Transient hypocalcaemia	132 (19·7)	134 (20·3)	266 (20·0)	0·743
Haematoma‡	6 of 669 (0·9)	10 of 658 (1·5)	16 of 1327 (1·2)	0·306
Time to haematoma (h)*	2·0 (0·2–6·0)	2·0 (1·0–3·3)	2·0 (1·0–4·0)	
Dysphonia	46 of 668 (6·9)	39 of 658 (5·9)	85 of 1326 (6·4)	0·476
Swallowing disorders	6 of 669 (0·9)	6 of 658 (0·9)	12 of 1327 (0·9)	0·977
Other complications	12 of 669 (1·8)	16 of 658 (2·4)	28 of 1327 (2·1)	0·401
RLN dysfunction	66 of 665 (9·9)	65 of 655 (9·9)	131 of 1320 (9·9)	0·996
Hypomotility	41	35	76	
Immobility	25	30	55	
Complications at 6 months				
RLN dysfunction	9 of 632 (1·4)	3 of 630 (0·5)	12 of 1262 (1·0)	0·101
Hypomotility	5	1	6	
Immobility	4	2	6	
Permanent hypocalcaemia	16 of 666 (2·4)	10 of 647 (1·5)	26 of 1313 (2·0)	–¶

Values in parentheses are percentages unless indicated otherwise;

* values are median (i.q.r.).

† Skin burning, oral haemorrhage.

‡ Compressive haematoma requiring surgical reoperation. RLN, recurrent laryngeal nerve.

§ Generalized linear mixed model.

¶ In non‐inferiority analysis, mean difference between conventional haemostasis and ultrasonic scissors groups = −0·9 (95 per cent c.i. −2·4 to 0·6) per cent.

The incidence of postoperative morbidity (transient or permanent hypocalcaemia, RLN palsy on day 2 and at 6 months) was also similar in the two arms, regardless of thyroid function (euthyroidism or hyperthyroidism), BMI (less than or at least 30 kg/m^2^), histology (benign or thyroid cancer), LND, duration of surgery or weight of the thyroid gland.

In total, 646 adverse events were reported in 479 patients, including 40 serious adverse events in 36 patients. After excluding RLN palsy and hypocalcaemia, the distribution of the 310 adverse events was: 250 grade I (134 US, 116 CH), 47 grade II (26 US, 21 CH), seven grade III (4 US, 3 CH) and six grade IV (3 US, 3 CH).

### Economic evaluation

Patients from one site were excluded because economic data could not be collected comprehensively. The mean(s.d.) duration of operating room occupancy was 152(52) (median 140, 120–180) min in the CH arm and 145(48) (135, 110–169) min in the US arm (P = 0·032)

Other main results of the FAS analysis are summarized in Table 3. The duration of operation was approximately 10 min shorter in the US arm, resulting in a significantly lower cost of human resources. Among the 13 participating sites, the median operating time ranged from 50 to 155 min in the US arm and from 50 to 140 min in the CH arm. The operating time had no impact on transient or definitive hypocalcaemia (P = 0·694) or transient RLN palsy (P = 0·173). The cost of devices and the total cost of the procedure were lower in the CH arm.

**Table 3:** Medicoeconomic data for full analysis set

	Ultrasonic scissors (n = 606)	Conventional haemostasis (n = 595)	P *
Duration of procedure (min)			< 0·001
Mean(s.d.)	97(39)	107(46)	
Median (i.q.r.)	90 (70–115)	100 (75–129)	
Initial length of stay (days)			0·853
Mean(s.d.)	2·7(0·9)	2·7(0·9)	
Median (i.q.r.)	3 (2–3)	3 (2–3)	
Cost of human resources (€)			< 0·001
Mean(s.d.)	463(174)	502(202)	
Median (i.q.r.)	431 (345–540)	462 (358–608)	
Operating room costs (€)			0·032
Mean(s.d.)	1148(388)	1199(422)	
Median (i.q.r.)	1066 (869–1335)	1106 (909–1422)	
Device cost (€)	565	265	< 0·001
Bed‐days cost (€)			0·594
Mean(s.d.)	1779(555)	1762(535)	
Median (i.q.r.)	1918 (1279–1918)	1918 (1279–1918)	
Total cost of procedure (€)			< 0·001
Mean(s.d.)	2176(555)	1966(616)	
Median (i.q.r.)	2065 (1803–2419)	1845 (1524–2294)	
Total cost of initial admission (€)			< 0·001
Mean(s.d.)	3954(792)	3728(831)	
Median (i.q.r.)	3865 (3433–4372)	3579 (3167–4160)	
No. of patients with ≥ 1 outpatient consultation	519 (77·4)	527 (86·7)	0·160
No. of consultations per patient			0·772
Mean(s.d.)	4·3(4·9)	4·3(4·6)	
Median (i.q.r.)	3 (2–5)	3 (2–5)	
No. of patients with repeat hospital admission	34 (5·6)	27 (4·5)	0·397
Cost of consultations and readmissions at 6 months (€)			0·323
Mean(s.d.)	357(1272)	283(1321)	
Median (i.q.r.)	84 (46–138)	84 (46–138)	
Time off work (days)			0·061
Mean(s.d.)	12(25)	15(31)	
Median (i.q.r.)	0 (0–15)	0 (0–19)	
Compensation for time off work (€)			0·059
Mean(s.d.)	457(1017)	584(1293)	
Median (i.q.r.)	0 (0–518)	0 (0–690)	
Total direct cost at 6 months (€)			< 0·001
Mean(s.d.)	4311(1547)	4011(1596)	
Median (i.q.r.)	4009 (3519–4535)	3702 (3255–4356)	
Total direct + indirect costs at 6 months (€)			0·143
Mean(s.d.)	4769(1970)	4595(2140)	
Median (i.q.r.)	4281 (3779–5084)	4122 (3520–5028)	

Values in parentheses are percentages unless indicated otherwise.

* Student's t or Mann–Whitney U test.

Mean length and cost of hospital stay were not significantly different between the CH and US arms. In the US arm, mean direct costs were higher but indirect costs (calculated from days of sick leave) were lower (P = 0·059) owing to a shorter duration of sick leave. The overall direct cost of US at 6 months was significantly higher but the difference was no longer statistically significant when indirect costs were added. The PP analyses identified a total initial mean(s.d.) cost of €3955(784) for US versus €3729(830) for CH, and a total cost of €4778(1981) and €4601(2148) respectively. P values were similar in the FAS and PP analyses.

**Figure 2:**
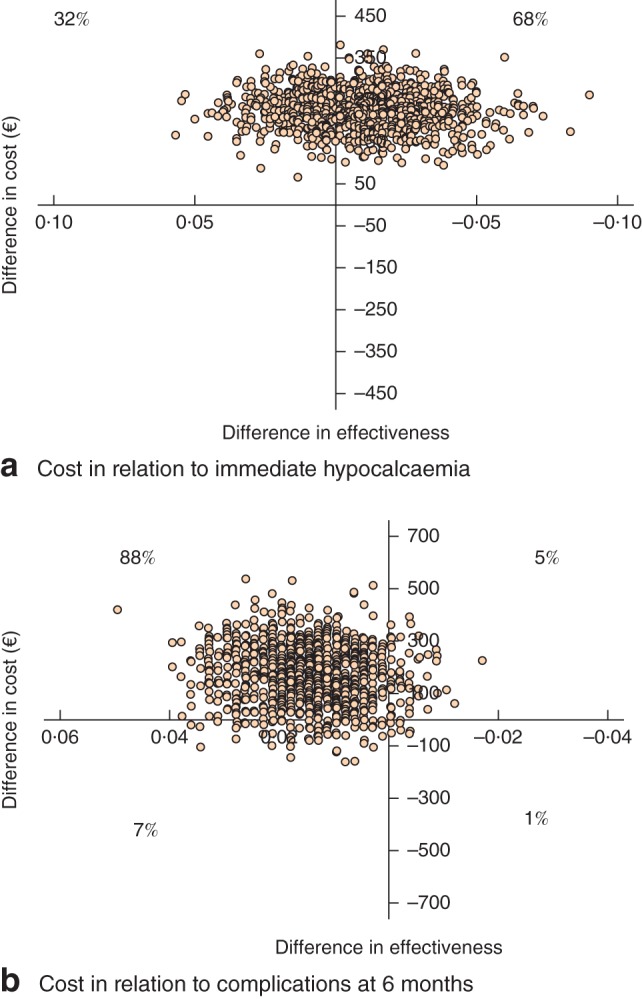
**a** Uncertainty associated with the immediate cost‐effectiveness of ultrasonic shears shown as a scatter plot of differences in mean cost and immediate hypocalcaemia between the two techniques. **b** Uncertainty associated with the 6‐month cost‐effectiveness of ultrasonic shears shown as a scatter plot of differences in mean cost and postoperative complications between the two techniques

Calculation of the incremental cost‐effectiveness ratio (ICER) for transient hypocalcaemia revealed that the mean cost for avoiding one case of hypocalcaemia was €15 427 (95 per cent c.i −126 774 to 124 477). At 6 months, US was less effective and more costly than CH. The deterministic sensitivity analysis identified length of hospital stay and operative costs as main factors influencing costs and the ICER. Fig. 2 shows the uncertainty associated with the cost‐effectiveness of US as a scatter plot of the difference in mean total cost relative to the difference in adverse events. There was a 68 per cent probability that US is more expensive but more effective in preventing immediate hypocalcaemia (Fig. 2 a), and an 88 per cent probability that US is more expensive and less effective in preventing adverse events at 6 months.

## Discussion

In this large multicentre RCT, there was no evidence of superiority of US over CH in decreasing immediate hypocalcaemia or other postoperative complications after total thyroidectomy. Despite a 10‐min decrease in operating time, the total cost of the US procedure was not lower, and the total direct costs at 6 months were higher than those of CH.

Previous studies of US in thyroid surgery were either small randomized trials or larger observational studies with varying inclusion criteria (Graves' disease or papillary carcinoma, other diseases). Although the ‘control arm’ can be standardized (for example using LigaSure™ (Valleylab, Boulder, Colorado, USA), bipolar forceps, knot and tie) or left at the discretion of the investigator, the latter was adopted in the present study; however, no centre employed LigaSure™ in the control arm.

Use of US has reduced the operating time in almost all published studies^17^, ^18^, ^19^, ^20^, ^21^, ^22^, ^23^, ^24^, ^25^, ^26^, ^27^, ^28^, ^29^, ^30^, including comparisons against LigaSure™. The present study confirmed that procedures in which US were used were quicker than those with CH. This represented the sole advantage of this device; a parameter that would be relevant only if it translated into clinical or economic benefits, which seemed less obvious.

Previous results with regard to hypocalcaemia have been discordant. In some studies^11^, ^20^, ^21^, ^25^, ^31^, transient hypocalcaemia was less common when US were used but, more often, rates were similar to those in the control arm^7^, ^8^, ^9^, ^18^, ^22^, ^23^, ^27^, ^29^, ^30^. Postoperative pain has rarely been evaluated, and only in a few studies^12^, ^21^, ^27^ did US reduce pain. The incidence of RLN palsy was also lower with US in some studies^18^, but more often has been comparable with that in the control arm^17^, ^20^, ^22^, ^30^, although the rate of this complication is so low that no study was large enough to detect differences.

Neither of the two large published cohort studies has shown major advantages in favour of US. Data from 1846 consecutive patients undergoing thyroidectomy using bipolar forceps or US, in a non‐randomized study^18^, indicated that the unadjusted risk of hypoparathyroidism was higher in women, in patients with thyroid cancer and if LND was undertaken, but with no difference between arms after adjustment for these confounding factors. In a randomized study comparing US with conventional clamp‐and‐tie haemostasis in 778 patients in China^20^, operating time was significantly lower with US (79 *versus* 125 min), and mean parathyroid hormone and calcium levels on day 1 were lower in the clamp‐and‐tie group, but the differences were not statistically significant and there was no difference in the rates of transient hypocalcaemia.

With regard to the cost‐effectiveness of US, only a few studies have performed detailed economic analyses. Some found the US device to be cost‐effective in terms of surgical procedure and/or hospital admission^24^, ^28^, whereas others^8^, ^9^ reported that, owing to the higher cost of disposable materials, the overall cost of hospitalization was not significantly different between US and CH groups. Economic analyses are difficult to extrapolate to different countries because of differences in hospital costs and organization. A French study^7^ compared US with a tie‐and‐clip procedure, and calculated a total cost of €1024 in the US group and €990 in the tie‐and‐clip group, with a non‐significant difference despite a shorter duration of surgery with use of US^7^. Of note, patients were not randomized, and allocation of the study group depended on the availability of the device. The present results are consistent with most previously published economic data, and confirm that lower hospital costs resulting from a shorter operating time do not compensate for the higher cost of devices.

The present study does have limitations. There was no RLN neuromonitoring, although there is no strong evidence that this influences the postoperative RLN palsy rate. The control arm (CH) was heterogeneous, with different surgical techniques used, so no inference can be made regarding US and individual CH techniques. No account was taken of the experience of investigators using US, although the technique is usually learned rapidly and its use does not modify the operating technique. All surgeons had used the US device in at least ten procedures before the trial, on the basis that learning curve for US was unlikely to exceed five procedures. Missing data were infrequent but, as might have been anticipated, mainly affected vocal cord examinations at 6 months. Sixty‐seven patients (38 US, 29 CH) refused nasofibroscopy at 6 months because their voice had recovered to normal levels or because they still had hoarseness and did not see any benefit of a second nasofibroscopy. The choice of hypocalcaemia as the primary endpoint may be debatable. It was chosen on the basis of its frequency compared with other complications that would have required far greater numbers of patients. The conclusions of the present study cannot be applied to patients with invasive cancers or substernal goitres, who were excluded.

The use of US, such as HARMONIC FOCUS^®^, did not decrease the incidence of transient hypocalcaemia or any other complication after total thyroidectomy, compared with CH, in the present study. As use of this device did not prove cost‐effective, within the limitations of the study, it is difficult to see how it can be advocated in preference to other techniques.

## Collaborators

FOThyr group members: V.‐P. Riche (Département Partenariats et Innovation, Cellule Innovation, Délégation à la Recherche Clinique et à l'Innovation (DRCI), Nantes); S. Mucci (Chirurgie Digestive et Endocrinienne, Centre Hospitalier Universitaire (CHU) Angers, Angers); C. Nominé (Service de Chirurgie Digestive, Hépato‐Biliaire et Endocrinienne, Nancy – Hôpital de Brabois, Nancy); R. Caiazzo (Chirurgie Générale et Endocrinienne, CHU Lille, Université de Lille, Lille); J. M. Prades (Oto‐Rhino‐Laryngologie (ORL) et Chirurgie Cervico‐Faciale et Plastique, CHU Saint‐Etienne – Hôpital Nord, Saint‐Etienne); G. Landecy (Chirurgie Digestive, CHU de Besançon – Hôpital Jean Minjoz, Besançon); H. P. Dernis (Service ORL et Chirurgie Cervico‐Faciale, Centre Hospitalier du Mans, Le Mans); J. C. Lifante (Chirurgie Générale, Endocrinienne, Digestive et Thoracique, Centre Hospitalier Lyon‐Sud, Pierre Bénite); F. Sebag (Chirurgie Générale, Assistance Publique des Hôpitaux de Marseille – Hôpital de La Conception, Marseille); F. Jegoux (Service ORL et Chirurgie Maxillo‐Faciale, CHU de Rennes – Hôpital Pontchaillou, Rennes); E. Babin (ORL et Chirurgie Cervico‐Faciale, CHU de Caen, Caen); A. Bizon (ORL et Chirurgie Cervico‐Faciale, CHU d'Angers, Angers); F. Espitalier, Service ORL, CHU de Nantes, Nantes).
